# Different Ultimate Factors Define Timing of Breeding in Two Related Species

**DOI:** 10.1371/journal.pone.0162643

**Published:** 2016-09-09

**Authors:** Veli-Matti Pakanen, Markku Orell, Emma Vatka, Seppo Rytkönen, Juli Broggi

**Affiliations:** 1 Department of Ecology, University of Oulu, P. O. Box 3000, FIN-90014 University of Oulu, Oulu, Finland; 2 Research Unit of Biodiversity, (UMIB, UO-CISC, PA). Ed. de Investigación 5ª C/ Gonzalo Gutiérrez Quirós s/n. 33600 Mieres, Spain; Universidad de Granada, SPAIN

## Abstract

Correct reproductive timing is crucial for fitness. Breeding phenology even in similar species can differ due to different selective pressures on the timing of reproduction. These selection pressures define species’ responses to warming springs. The temporal match-mismatch hypothesis suggests that timing of breeding in animals is selected to match with food availability (synchrony). Alternatively, time-dependent breeding success (the date hypothesis) can result from other seasonally deteriorating ecological conditions such as intra- or interspecific competition or predation. We studied the effects of two ultimate factors on the timing of breeding, synchrony and other time-dependent factors (time-dependence), in sympatric populations of two related forest-dwelling passerine species, the great tit (*Parus major*) and the willow tit (*Poecile montanus*) by modelling recruitment with long-term capture-recapture data. We hypothesized that these two factors have different relevance for fitness in these species. We found that local recruitment in both species showed quadratic relationships with both time-dependence and synchrony. However, the importance of these factors was markedly different between the studied species. Caterpillar food played a predominant role in predicting the timing of breeding of the great tit. In contrast, for the willow tit time-dependence modelled as timing in relation to conspecifics was more important for local recruitment than synchrony. High caterpillar biomass experienced during the pre- and post-fledging periods increased local recruitment of both species. These contrasting results confirm that these species experience different selective pressures upon the timing of breeding, and hence responses to climate change may differ. Detailed information about life-history strategies is required to understand the effects of climate change, even in closely related taxa. The temporal match-mismatch hypothesis should be extended to consider subsequent critical periods when food needs to be abundantly available.

## Introduction

Correct timing of reproduction is crucial in seasonal environments [1–3]. Ecological factors affecting the timing of reproduction as well as the underlying physiological mechanisms have been widely studied, particularly since climate warming has caused many species to advance their reproduction [4–5]. However, reproductive phenologies of even very similar species as well as their responses to climate warming are not fully understood [5].

Reproductive timing is affected by many selective pressures acting on different life history stages [6–7]. The temporal match-mismatch hypothesis suggests that timing of breeding in animals is selected to match with food availability so that the timing of the highest food demand of offspring is synchronous with the food availability peak [8], resulting in stabilizing selection [9]. Ongoing climate change has rapidly changed phenologies at lower trophic levels, sometimes leading to temporal mismatches that have caused directional selection pressures on the timing of reproduction of their predators [10–11].

Alternatively, time-dependent breeding success can also cause directional selection for earlier breeding (the date hypothesis, [12–13]); a universal phenomenon [14–15] mainly caused by seasonally deteriorating conditions [14, 16]. Time-dependence is particularly important for resident species with intraspecific competition for territories and dominance positions [9]. Earlier born individuals benefit from prior residency, better access to territory ownership and dominant positions in conspecific winter flocks [17–18]. These benefits enhance their survival prospects in relation to late born young [17–18]. Therefore, timing of breeding with respect to conspecifics is of major importance in species that have territorial groups with social hierarchy [17–18], and may outweigh the benefits of a temporal match with abundant food resources. Interspecific competition for resources (e.g. nesting sites or food) is another time-dependent factor that may cause species with low competition abilities to benefit from breeding early [19]. Also, the predation escape hypothesis [20] predicts directional selection for earlier breeding if early breeding individuals and their offspring avoid the highest predation pressure either during the breeding event or in later life-history stages (e.g. during migration).

Much of the work on the ecological consequences of climate change has focused on the shifts in the timing of breeding following warming spring temperatures, trophic mismatches and resulting effects on breeding success and population size [2–3, 7–11, 21–24]. Yet, effects of other time-dependent selective pressures on the current evolution of reproductive timing have so far received much less attention. Differences in other life history stages can strongly affect species’ responses to warming springs [9], even in coexisting closely related species [25–26]. Comparison of sympatric populations of closely related species that differ in some aspects of life history can increase our understanding about the mechanisms underlying these different responses.

We studied the ultimate factors on the timing of breeding: phenological mismatch and time-dependence, in sympatric populations of two related forest-dwelling passerine species in northern Finland, the great tit (*Parus major*, L.) and the willow tit (*Poecile montanus*, Conrad). Both species have temporally advanced their breeding along with the availability of their main prey, insect larvae [21–22]. However, these species differ in the timing of breeding yet they rely on the same main food source (insect larvae) during the nestling stage [27]. The great tits time their breeding so that the period of the highest food needs of the nestlings (around the age of 10 days) co-occurs with the period of the highest food availability i.e. caterpillar peak [22]. Among Parids, such timing of breeding is considered being in good synchrony, a phenological match [10]. In contrast, boreal willow tits often breed earlier and in poor synchrony with caterpillar food [21]. We therefore hypothesize that the two ultimate factors affecting the timing of breeding have different relevance for fitness in two study species. We tested this with long-term capture-recapture data (1999–2012) by modelling recruitment, the life history stage that ultimately determines fitness [14]. The results confirmed that local recruitment of great tits was strongly dependent on caterpillar food, whereas in the willow tit, local recruitment depended on the timing of conspecifics, not caterpillar food.

## Materials and Methods

### Field methods

The data were collected in Oulu, Finland (N 65°08', E 25°53') between 1999 and 2012. The study area contains coniferous, deciduous and mixed forests of varying ages and is a part of a continuous managed forest landscape (S1 Fig). The main trees include birch (*Betula* sp.), Scotch pine (*Pinus sylvestris*) and spruce (*Picea abies*) [28]. The willow tit study area is uniform, roundish shaped and ca. 25 km^2^ in size (S1 Fig). The great tit study area (ca. 8 km^2^) overlaps that of the willow tits, but is very different in shape (S1 Fig). The number of nest boxes was ca. 400 throughout the study period. Intensive field routines ensured that almost all nests were found and all individuals were ringed [22]. We started searching for territories and nests in early-April. We recorded dates when laying started and visited the nests until clutches were complete. Average clutch sizes are 7.6 eggs for willow tits [29] and 10 for great tits [30]. Then, we used the incubation time of ca 14 days (both species; [29–30]) to estimate time of hatching, which was confirmed by frequent visits around the estimated hatching date. Nestlings were marked with aluminium rings when 13–15 days old, before fledging which occurs at the age of 18 days [30–31]. Recruitment was recorded by recapturing parent birds at their nests in subsequent years. All adults were ringed with individual colour codes. All field procedures adhered to Finnish national legislation. Trapping and ringing of animals was done under license from the Finnish Natural History Museum in Helsinki. This work did not involve invasive sampling that requires permits from the National Animal Experiment Board. Field studies were performed on private and government lands. People have the right to access the Finnish land areas freely under the traditional Finnish legal concept known as everyman's right unless specifically protected or prohibited.

In the study populations, Parids mostly forage on caterpillars (e.g. *Epirrita autumnata*) occurring in birches (*Betula* spp.) during the breeding season [27–28]. The frassfall method was used to estimate caterpillar availability [32]. The quantity of caterpillar droppings falling on a certain area (m^2^) per unit of time (day) was used to estimate the biomass of caterpillars foraging on birch for weekly periods. We used five collectors (one per tree) in four different places (20 collectors) to control for spatial variation in caterpillar phenology within the study area. The annual peak dates of caterpillar abundance were determined to be the middle day of a period when the biomass was at its highest. However, the annual peaks occurred almost always in the same week in different locations. Highly synchronous caterpillar phenology is a consequence of the very synchronous bud burst of birch in the study area; supposedly as this is a very flat area (15–30 m above sea level). Therefore, a single caterpillar peak date was employed for the entire study area.

### Data analyses

Apparent juvenile survival from fledging to one year of age (i.e. local recruitment) was analysed from marked fledglings (*P*. *major*: 6294 individuals, *P*. *montanus*: 7224 individuals) born in first, replacement and second nests. Survival was modelled using CJS-models in program MARK 8.0 [33–34]. Global models considering time and age structure in survival, and time in the recapture probabilities [i.e. Phi (age*t) p(t)] fit both data (GOF_PARAMETRIC BOOTSTRAP_: *major*: p = 0.213, ĉ = 1.137; *montanus*: p = 0.364, ĉ = 1.039).

The timing in relation to the caterpillar peak was defined with a variable ‘synchrony’ as the difference between the day when the nestling was 10 days old and the caterpillar peak date (synchrony = hatching date + 10 –caterpillar peak date; [21–22]. A value of zero indicates a perfect match. Timing in relation to the population mean was modelled with the year centred hatching date (‘HD’ = hatching date–annual mean hatching date). For both variables, linear and quadratic terms were tested. The correlation coefficients between ‘synchrony’ and ‘HD’ were 0.82 for the great tit and 0.5 for the willow tit. We first constructed competing models including either the 'synchrony' or 'HD' variable. When the best model for each species was defined (with either 'synchrony' or 'HD'), we tested if the inclusion of linear and quadratic terms of the less important variable improved the model fit.

Other variables included in the analysis were autumn population density (‘density’ = (number of parents + fledglings) / study area), both linear and quadratic terms of fledgling mass (‘mass’, g and ‘mass2‘). Models with ‘synchrony’ included ‘peak height’ (the maximum value of caterpillar biomass). We first fitted models with different combinations of the main effects only. We selected the three best main effect models and continued the model selection by including interactions. Only biologically interesting interactions with ‘synchrony’ or ‘HD’ were included. Models had a maximum of two interactions. See S1 Table for the numerical summaries of explanatory variables.

The importance of food availability encountered by the nestlings was also examined by including daily mean caterpillar biomass (BM). Biomass was measured for the whole pre-fledging period (‘BM1’ = caterpillar biomass at the ages of 0–18 days), the period of the highest food needs (‘BM2’ = caterpillar biomass at the ages of 8–13 days) and the post-fledging period (‘BM3’ = caterpillar biomass at the ages of 18–25 days). Biomass was modelled with and without ‘HD’. This analysis included only those individuals for which we had sufficient data on caterpillar abundance. The global models, [i.e. Phi(age*t) p(t)], fit both data (great tits: p = 0.06, ĉ = 1.27; willow tits: p = 0.38, ĉ = 1.03).

We accounted for possible bias arising from dispersal patterns in relation to the study area boundaries [35] by constraining both juvenile survival and recapture probabilities with distance from the natal nest to the centre of the study area (‘DC’) and checked for interactions between ‘DC’ and the ‘HD’ to test for date dependent emigration. If an interaction was found, survival was predicted for near the centre of the study area (DC = 1000 m) to control for date dependent emigration.

### Model selection

We used AIC in model selection [36]. We first examined relative support for variables ‘synchrony’ and ‘HD’ using cumulative Akaike weights. Because ‘synchrony’ was included in more models than ‘HD’ due to variable ‘peak height’ we averaged the cumulative QAIC-weights. When predicting the responses to synchrony, we first examined if the best model containing the variable of interest (either ‘synchrony’ or ‘HD’) received a better model fit (i.e., smaller QAIC) when the other competing factor and its quadratic term where included in the model. This was done when predicting the response to synchrony in order to control for the effect of timing in relation to others. Uncertainty in model selection was considered by calculating weighted average survival estimates from models with ΔQAIC <2 [36]. We present regression parameter estimates from the most supported models containing the variable unless mentioned otherwise. The parameters in the supplementary tables are presented on the logit scale.

## Results

### The great tit

**Fig 1:**
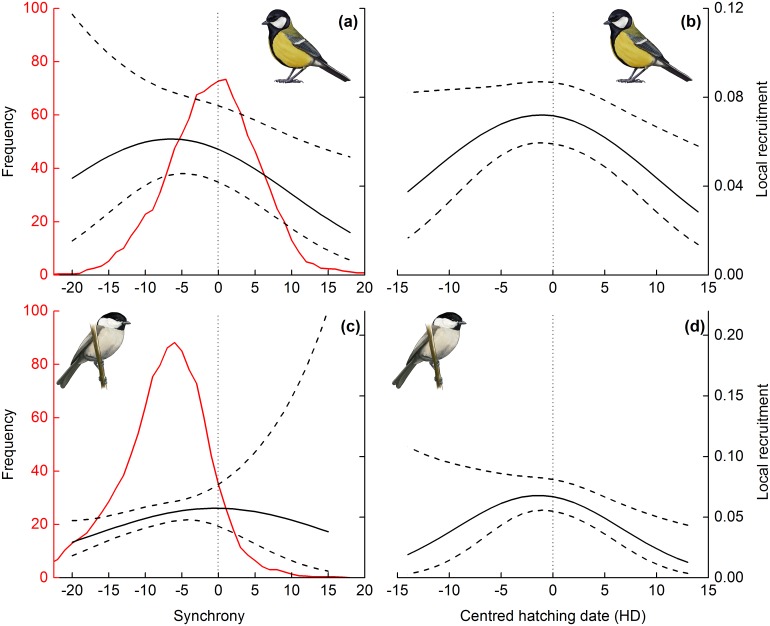
Local recruitment and the timing of great tits and willow tits. The local recruitment of the great tit (a and b) and the willow tit (c and d) in relation to synchrony with the caterpillar food peak (synchrony = day of 10 days old young–caterpillar peak day, i.e. chicks are 10 days old at the peak day; a and c), and local recruitment in relation to the timing of conspecifics (b and d). Frequency distribution for the observed synchrony values (a sliding average of five days) is shown by a red line in a and c. In d, date dependent emigration is controlled by an interaction between centred hatching date and distance to centre (1000m in this figure). Dashed lines indicate 95% confidence intervals for local recruitment. The scale changes between rows. Parameter coefficients are presented in S7–S10 Tables (Supplementary material).

**Fig 2:**
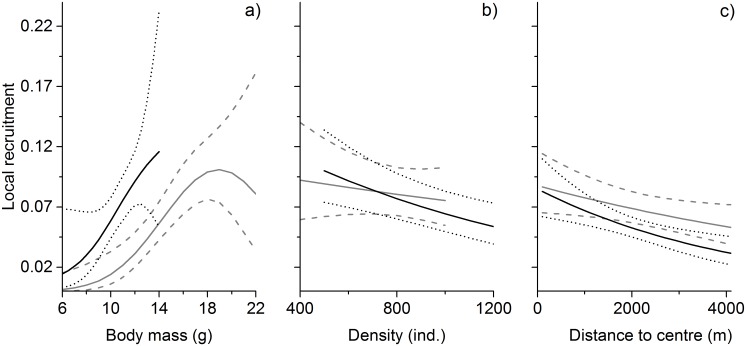
Variation in local recruitment of willow tits and great tits. Effects of a) body mass, b) density and c) distance to the centre of the study area from the natal site on local recruitment of willow tits (black solid line, dotted 95% confidence intervals) and great tits (grey solid line, dashed 95% confidence intervals).

Great tits bred in good synchrony with the food peak (Fig 1a). The local recruitment of great tits showed a quadratic relationship with synchrony (S2, S4, S5 and S7 Tables). When hatching date was controlled for (S5 Table), local recruitment was highest when chicks were 15 to 19 days old during the food peak (Fig 1a). Peak height increased local recruitment but it did not interact with synchrony (S2, S4 and S7 Tables). Synchrony was 3.4 times more relevant to recruitment than the centred hatching date (S4 Table). Hatching date also had a quadratic effect on local recruitment (S2, S4 and S8 Tables, Fig 1b). Both synchrony and hatching date interacted with density (S1, S4, S7 and S8 Tables). The interaction was especially strong between density and both linear and quadratic terms of synchrony. In high densities, early birds had higher survival but in low densities birds that were late in relation to the food peak or conspecifics had higher survival prospects (S2 Fig). Fledgling mass had a strong influence on local recruitment and distance to the centre of the study area showed a negative but non-significant trend (S2, S4, S7 and S8 Tables; Fig 2).

Caterpillar biomass during the fledgling period (when the young were 18–25 days old) increased local recruitment in great tits (S11 and S13 Tables). Its effect was not dependent on whether or not hatching date was included in the model (S11 and S13 Tables). However, caterpillar biomass accumulated during the whole nestling period (ages 0–18 days) or during the time considered being of the highest food demand (ages 8–13 days), had minor effects on local recruitment (S11 and S13 Tables).

### The willow tit

**Fig 3:**
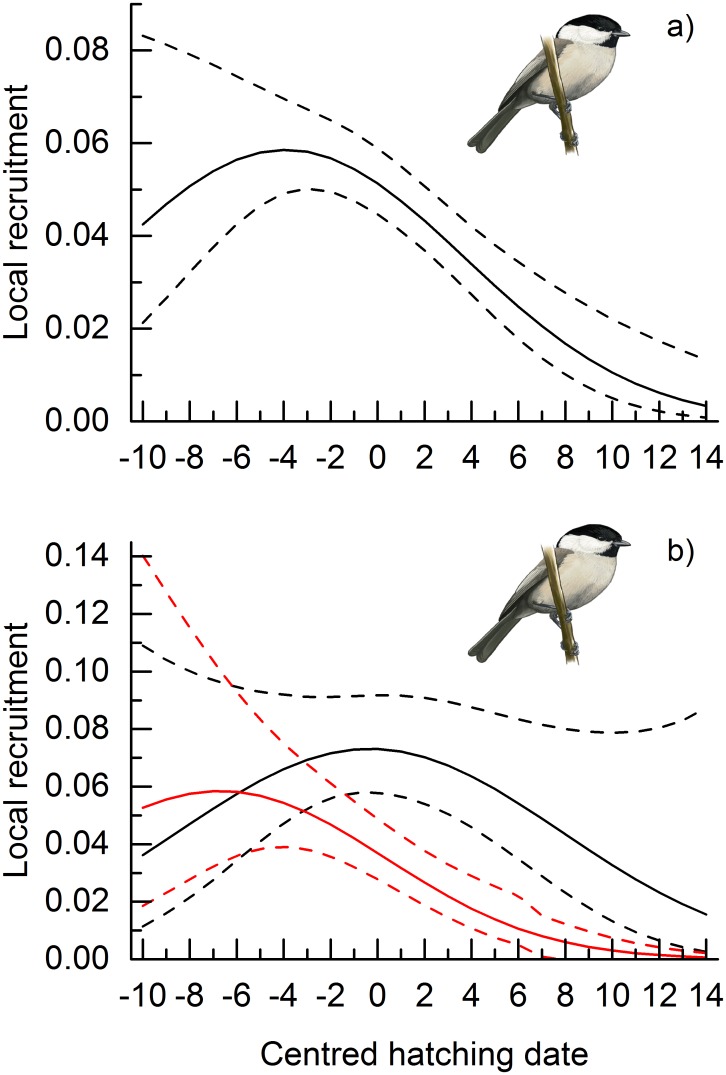
Date dependent local recruitment in the willow tit. Local recruitment in relation to the timing of conspecifics a) when considering only a standalone effect, and b) when considering interaction between centred hatching date (linear and quadratic) and distance to the centre of the study area (‘DC’; red line, DC = 3500; black line DTC = 500, dashed lines indicate 95% confidence intervals).

Willow tits bred on average 7 to 8 days earlier than the occurrence of the food peak (Fig 1c). Local recruitment was strongly affected by hatching date (S3, S4 and S10 Tables), a standalone effect suggesting strong directional selection for early breeding (Fig 3a). However, distance to the centre of the study area had a negative impact on local recruitment (S3, S4 and S10 Tables; Fig 2) indicating that individuals born at the edge of the study area had higher probability of emigration. Furthermore, distance to the centre of the study area interacted with both linear and quadratic terms of hatching date (S3, S4 and S10 Tables; Fig 3). The interaction means that for individuals that originated at the edge of the study area, hatching early was extremely important for recruitment into the study area (Fig 3b). However, for birds that originated close to the centre of the study area, average hatching resulted in the highest local recruitment (Fig 3b). These interactions suggest date-dependent dispersal, which should be accounted for. We thus used a distance of 1000 metres from the centre of the study area to derive model predictions (i.e. controlling for emigration), that suggested the highest local recruitment to be achieved by hatching one to three days before average (Fig 1d).

The hatching date was 5,718.0 times more important than synchrony when explaining local recruitment. Synchrony increased model support when included with the best model with hatching date (S6 Table). Nevertheless, its impact remained small (S9 Table). When controlling for hatching date (= 0), local recruitment was the highest when synchrony was good, i.e. chicks were 10 days old (Fig 1c). Local recruitment was also affected by mass and density but no interactions with hatching date or synchrony were found (S3, S4 and S10 Tables; Fig 2).

The effect of caterpillar biomass on local recruitment was dependent on the hatching date. If hatching date was included, caterpillar biomasses of all periods considered increased local recruitment (S12 and S14 Tables). The effect was particularly strong for ages 0–18 days and 8–13 days (ΔAIC ≥ 10; S12 and S14 Tables). If hatching date was not considered, caterpillar biomass did not seem to affect recruitment.

## Discussion

We found that local recruitment in both species showed quadratic relationships with both timing and synchrony, supporting the view of an optimal timing of breeding [6, 14]. However, the importance of these factors was strongly different. As expected, caterpillar food played a predominant role in predicting the timing of breeding of the great tit–synchrony explained recruitment 3.4 times better than timing. In contrast, for the willow tit, synchrony was outweighed by timing related factors, with timing explaining local recruitment 5,718.0 times better than synchrony. High caterpillar biomass experienced during the pre- and post-fledging periods increased the local recruitment of both species. These contrasting results suggest that different life history strategies of these species result in differential selection pressures acting on the timing of breeding, and hence responses to climate change may differ.

### The relevance of temporal match with the food peak

Despite the apparent good timing of the great tit with caterpillar prey [21], we found that local recruitment was the highest in broods in which the young were 15–19 days old when the food availability peaked, i.e., at the time of fledging (Fig 1a). In accordance, caterpillar biomass during the post-fledging stage (i.e. ages 18–25) was the most influential for local recruitment. Thus, the commonly used measure for ‘good synchrony’ (temporal match between the time of the highest food demand of the nestlings at the age of 10 days and the highest food availability) may not be universal. The temporal match-mismatch hypothesis should be extended to consider several critical periods when high-quality food needs to be abundantly available–not only the period of the fastest growth of the offspring, but also the time of newly fledged young learning to forage independently–for the breeding event to be successful.

The great tit thus appears to breed too late to maximize local recruitment in the northern conditions (Fig 1a). Originally a temperate species, great tits have shown the ability to advance breeding [22], but may be unable to start breeding at the time that would maximize local recruitment in the northern conditions. Early breeding may be constrained by possible carry-over effects from wintering, high energetic requirements of egg-laying and incubation or food shortage during the nestling stage [24, 37]. Thus, the realized timing may be the most optimal possible solution [6].

The effect of synchrony (but less that of timing) on the local recruitment of the great tit depended on population density (S2 Fig). In high densities, early breeding in relation to the caterpillar peak resulted in the highest survival. Therefore, conspecific competition between young may select for earlier breeding, and partly explain the lack of association between peak food demand (10 days old chicks) and highest survival. As the early fledged young are presumably dominant over late fledging young, early fledged young may have higher survival during the period just after gaining independence [38]. These results are congruent with [39] who found stronger selection on early laying date with increasing population density in a great tit population. This effect may result from differences in quality of territories or from early hatching young faring well in competition for food later in their first autumn and winter [39]. It is also possible that high densities result from good conditions (e.g. the abundance of other food sources or weather) that favour survival. Nevertheless, it seems that selection on the timing of breeding is stabilizing at average or lower densities when conditions may be less favourable (Fig 1b).

Willow tits timed their breeding so that the young fledged on average at the time of the highest caterpillar availability (Fig 1c). Interestingly, while such synchrony maximized local recruitment for the great tit, local recruitment for the willow tit appeared the highest when the chicks were ca. 10 days old at the time of the highest caterpillar availability (Fig 1c). This is consistent with theory on phenological matching in Parids [10]. In addition, high caterpillar biomass during the nestling stage (but not post-fledging) increased local recruitment but only when timing in relation to conspecifics was considered. The willow tit therefore breeds before the peak of food abundance despite the fact that high caterpillar availability is beneficial for the survival of the young. Such behaviour is apparently adaptive because of other factors affecting the timing of breeding [40–41].

### Strong directional selection or date dependent dispersal?

As a standalone effect, time-dependence indicated strong directional selection for early breeding in the willow tit (Fig 3a). However, distance to the centre interacted with the timing variables. Near the edge of the study area, survival rates declined strongly in time as if there was strong directional selection for early breeding, but at the centre of the study area, where the effect of emigration is smaller, average timing resulted in the highest survival (Fig 3b). After controlling for such date dependent dispersal, it seems that selection is more or less balancing (Figs 1d and 3b). This result is more realistic because without the correction the results would suggest that there are no costs from demanding early season conditions, which were apparent to the great tit.

### Time-dependent selection pressures

Strong social influences in the willow tit are one explanation for the overwhelming importance of timing breeding in relation to conspecifics for local recruitment. Wintering strategy of willow tits involves hoarding food and spending the non-breeding season in their future breeding grounds in highly territorial and coherent non-kin groups, in which both access and dominance ranks are determined by prior residency [18]. It is known that late fledging individuals are in a poor situation in terms of intra-specific competition for attaining high dominance positions in the wintering groups and hence survival over the winter (*P*. *palustris*, [17]; *P*. *montanus*, [18, 42]). This together with strong density dependent local recruitment found in this study suggests that the wintering strategy ultimately affects the timing of breeding via prior residency effects on local recruitment [9, 18, 42]. However, we note that while it is important for willow tits to breed in relation to conspecifics this mechanism does not seem to result in strong selection for early breeding (Fig 1d), rather they should not breed too late.

This view is supported by ecologically similar species that winter in small flocks with strong social hierarchies and food hoarding, and also breed early in relation to caterpillar food (e.g. the marsh tit (*P*. *palustris*; [17, 43–45], the Siberian tit (*P*. *cincta*; [46], the crested tit (*Lophophanes cristatus*; [47–48]). Most temperate and boreal Parids show similar social systems linked with food hoarding during winter [49]. Interestingly, great tits and blue tits (*Cyanistes caeruleus*) time their breeding to match with caterpillar food, and are exceptions to this rule [12, 50–52]. In the boreal zone, they winter in constantly changing non-territorial flocks in areas that may be located far from their future breeding territories, and while prior residence affects dominance relationships, it does not dictate access to wintering groups [49, 53–54]. Thus, a possible explanation for the lower importance of timing breeding in relation to conspecifics in the great tit may be linked to different adaptations for wintering.

Although the relevance of social influences during the non-breeding season on the timing of breeding is a reasonable explanation, our results do not strictly confirm this mechanism to be the ultimate one for the different fitness responses. We cannot exclude interspecific competition for nesting sites [31] or for food during the nestling period [19], both of which may result in temporal niche separation, that is, phenological differences in the timing of breeding. Poor competitors may escape competition by breeding earlier than their competitors, gaining higher breeding success than when breeding in synchrony with the competitors. Likewise, early breeding may result in a temporal escape from a high predation pressure [20, 31]. Both of these factors may function as mechanisms for time-dependent, directional selection pressures.

In migratory species, selection pressures related to migratory behaviour can constrain the timing of breeding [55] and therefore they are likely to show higher levels of mismatch with food availability than resident species. Our results suggest that a similar pattern of divergent ultimate factors in the timing of breeding can be found also among resident, related species. A certain breeding/wintering strategy is possible only if species’ other life history characteristics permit it. For example, the willow tit can use its winter food caches as an energy resource for egg production and a more diverse diet for providing their young [21, 27]. Thus, it can breed early with its breeding success being less conditional on the phenological match with the caterpillar peak than in the great tit.

Natural selection operates on the timing of breeding via several ultimate factors. Our species comparison provides evidence for the importance of life history differences in responses to the climate change. One life history characteristic, e.g. the wintering strategy or the competition ability, can function as a leverage point for the evolution of other characteristics, including the timing of breeding. Thus, detailed information on life histories is required when trying to understand the evolutionary and ecological consequences of climate change.

## Supporting Information

S1 Fig Map of the study area. The great tit study area is shown with the location of the boxes and the boundaries of the willow tit study area are depicted by a line drawn from the outermost nests observed during the study.
(PDF)

S2 Fig Effects of density and synchrony to local recruitment of great tits. Local recruitment of the great tit in relation to synchrony with the caterpillar food peak (synchrony = day of 10 days old young–caterpillar peak day, i.e. chicks are 10 days old at the peak day) in high densities (black lines; 940 individual / study area) and in low densities (red lines; 488 individuals / study area). Other variables were set to average values. Dashed lines indicate 95% confidence intervals. See S7 Table for model parameter coefficients.
(DOCX)

S1 Table Summaries of explanatory variables. (DOCX)

S2 Table Modelling results for local recruitment of great tits. Modelling results for local recruitment of the great tit (*Parus major*) survival examining the effects of centred hatching date (HD) and synchrony (SYN). Models also include PK = peak height in caterpillar food abundance, DC = distance to the center of the study area, MASS = mass, DEN = density, + additive effects, *interaction and variable name2 = quadratic effect of the variable, k = number of parameters. QAIC is scaled with ĉ = 1.137. Model parameters for survival include the intercept and age, and for recapture rates the intercept, but model names include only the covariates to increase readability.
(DOCX)

S3 Table Modelling results for local recruitment of willow tits. Modelling results for local recruitment of the willow tit (*Poecile montanus*) examining the effects of centred hatching date (HD) and synchrony (SYN). Models also include PK = peak height in caterpillar food abundance, DC = distance to the center of the study area, MASS = mass, DEN = density, + additive effects, *interaction and variable name2 = quadratic effect of the variable. QAIC is scaled with ĉ = 1.039. Model parameters for survival include also the intercept and age, and for recapture rates the intercept and time, but model names include only the covariates to increase readability.
(DOCX)

S4 Table Relative importance of variables. Relative importance of variables from models examining the effects of synchrony and hatching date on local recruitment (S2 and S3 Tables) shown by summed model weights (w) and average model weights (n = number of models) for both the great tit and the willow tit.
(DOCX)

S5 Table Modelling results for local recruitment of the great tit with synchrony and hatching date in the same model. Modelling results for local recruitment of the great tit (*Parus major*) examining the effects of and synchrony (SYN) when centred hatching date (HD) and its quadratic term (HD2) are included in the best model from S2 Table. Models also include PK = peak height in caterpillar food abundance, DC = distance to the center of the study area, MASS = mass, DEN = density, + additive effects, *interaction and variable name2 = quadratic effect of the variable, k = number of parameters. QAIC is scaled with ĉ = 1.137. Model parameters for survival include the intercept and age, and for recapture rates the intercept, but model names include only the covariates to increase readability.
(DOCX)

S6 Table Modelling results for local recruitment of the willow tit with synchrony and hatching date in the same model. Modelling results for local recruitment of the willow tit (*Poecile montanus*) examining the effect of synchrony (SYN) when synchrony (SYN) and its quadratic term (SYN) are included in the best model from S3 Table (i.e. a model containing centred hatching date). Models also include DC = distance to the center of the study area, MASS = mass, DEN = density, + additive effects, *interaction and variable name2 = quadratic effect of the variable. QAIC is scaled with ĉ = 1.039. Model parameters for survival include also the intercept and age, and for recapture rates the intercept and time, but model names include only the covariates to increase readability.
(DOCX)

S7 Table Regression coefficients on the top models describing great tit local recruitment in relation to synchrony. The regression coefficients (SE) for the top models (Delta QAICc <2) describing local recruitment of the great tit in relation to synchrony from S5 Table. Coefficients are presented in the logit scale. Variables that had confidence intervals that do not include zero are in bold.
(DOCX)

S8 Table Regression coefficients on the top models describing great tit local recruitment in relation to hatching date. The regression coefficients (SE) for the top models (Delta QAICc <2) describing local recruitment of the great tit in relation to centred hatching date from S2 Table. Coefficients are presented in the logit scale. Variables that had confidence intervals that do not include zero are in bold.
(DOCX)

S9 Table Regression coefficients of the top models describing great tit local recruitment in relation to synchrony. The regression coefficients (SE) for the top models (Delta QAICc <2) describing local recruitment of the willow tit in relation to synchrony from S6 Table. Coefficients are presented in the logit scale. Variables that had confidence intervals that do not include zero are in bold.
(DOCX)

S10 Table Regression coefficients of the top models describing great tit local recruitment in relation to hatching date. The regression coefficients (SE) for the top models (Delta QAICc <2) describing local recruitment of the willow tit in relation to centred hatching date from S3 Table. Coefficients are presented in the logit scale. Variables that had confidence intervals that do not include zero are in bold.
(DOCX)

S11 Table Modelling results for local recruitment of the great tit in relation to available caterpillar biomass. Modelling results for local recruitment of the great tit (*Parus major*) examining the effects of available caterpillar biomass (BM1 = during ages 0–18; BM2 = during ages 8–13; BM3 = during ages 18–25). The models also include HD = the centred hatching date, DC = distance to the center of the study area, MASS = mass, DEN = density, + additive effects, *interaction and variable name2 = quadratic effect of the variable. QAIC is scaled with ĉ = 1.27. Model parameters for survival include also the intercept and age, and for recapture rates the intercept and time, but model names include only the covariates to increase readability.
(DOCX)

S12 Table Modelling results for local recruitment of the willow tit in relation to available caterpillar biomass. Modelling results for local recruitment of the willow tit (*Poecile montanus*) survival examining the effects of available caterpillar biomass (BM1 = during ages 0–18; BM2 = during ages 8–13; BM3 = during ages 18–25). The models also include HD = the centred hatching date, DC = distance to the center of the study area, MASS = mass, DEN = density, + additive effects, *interaction and variable name2 = quadratic effect of the variable. QAIC is scaled with ĉ = 1.03. Model parameters for survival include also the intercept and age, and for recapture rates the intercept and time, but model names include only the covariates to increase readability.
(DOCX)

S13 Table Regression coefficients of the top models describing great tit local recruitment in relation to caterpillar biomass. The regression coefficients (β) and SE for models describing local recruitment of the great tit derived from models E1, E3 and E5 in S11 Table. Coefficients are presented in the logit scale. Variables that had confidence intervals that do not include zero are in bold.
(DOCX)

S14 Table Regression coefficients of the top models describing willow tit local recruitment in relation to caterpillar biomass. The regression coefficients (β) and SE for models describing local recruitment of the willow tit derived from models F1, F2 and F3 in S12 Table. Coefficients are presented in the logit scale. Variables that had confidence intervals that do not include zero are in bold.
(DOCX)
