# Supplementary material for: Different Ultimate Factors Define Timing of Breeding in Two Related Species
Source: PLoS One. 2016 Sep 9;11(9):e0162643. doi: 10.1371/journal.pone.0162643 (PMC5017718; doi:10.1371/journal.pone.0162643)
Supplement: S1 Fig — The great tit study area is shown with the location of the boxes and the boundaries of the willow tit study area are depicted by a line drawn from the outermost nests observed during the study. (PDF) [file pone.0162643.s001.pdf]

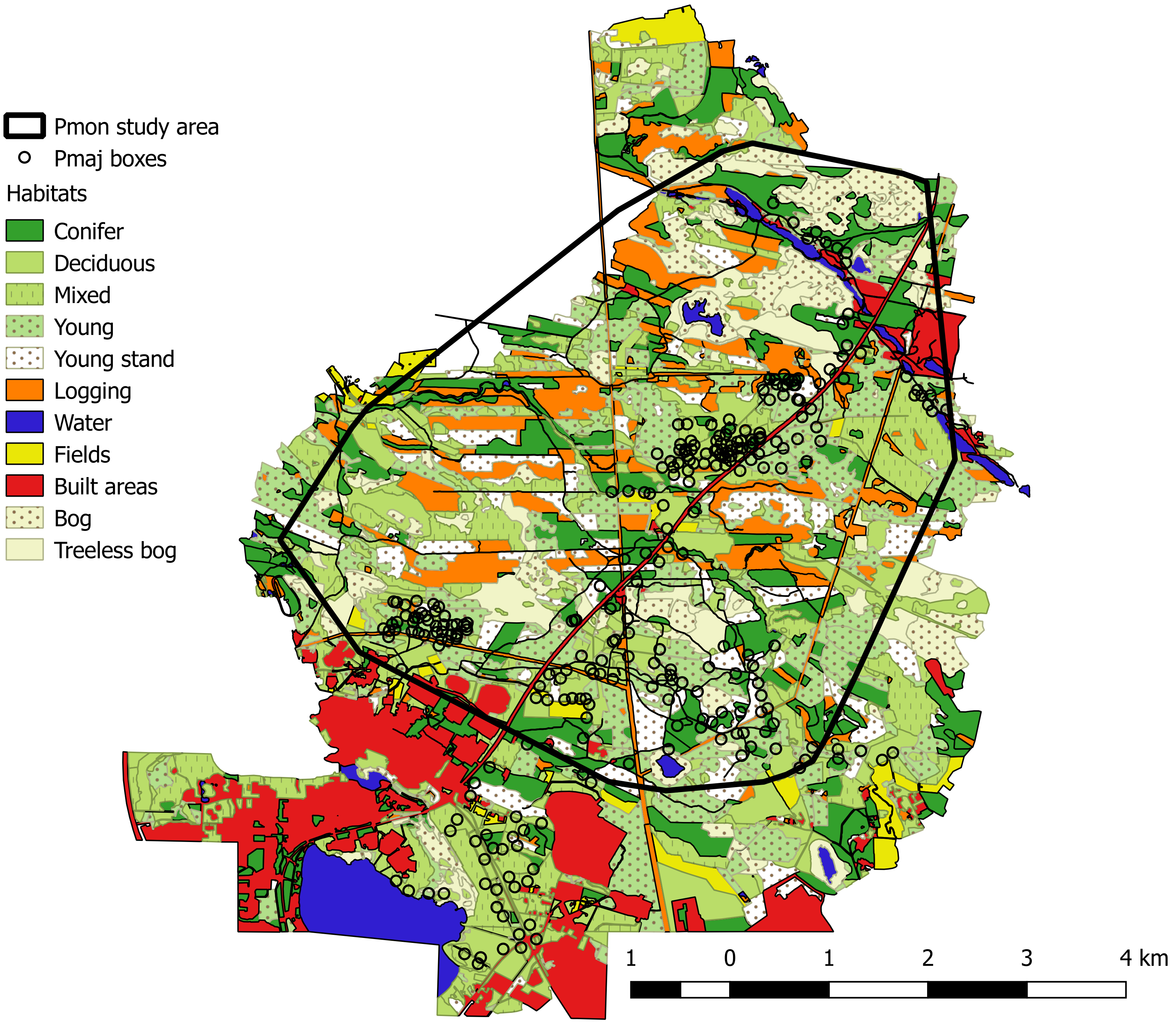
Pmon study area
Pmaj boxes
Habitats
Conifer
Deciduous
Mixed
Young
Young stand
Logging
Water
Fields
Built areas
Bog
Treeless bog
1
0
1
2
3
4 km
