# Supplementary material for: Different Ultimate Factors Define Timing of Breeding in Two Related Species
Source: PLoS One. 2016 Sep 9;11(9):e0162643. doi: 10.1371/journal.pone.0162643 (PMC5017718; doi:10.1371/journal.pone.0162643)
Supplement: S2 Fig — Local recruitment of the great tit in relation to synchrony with the caterpillar food peak (synchrony = day of 10 days old young–caterpillar peak day, i.e. chicks are 10 days old at the peak day) in high densities (black lines; 940 individual / study area) and in low densities (red lines; 488 individuals / study area). Other variables were set to average values. Dashed lines indicate 95% confidence intervals. See S7 Table for model parameter coefficients. (DOCX) [file pone.0162643.s002.docx]

**S2 Fig. Effects of density and synchrony to local recruitment of great tits.**

Different ultimate factors define timing of breeding in two related species

Veli-Matti Pakanen, Markku Orell, Emma Vatka, Seppo Rytkönen & Juli Broggi

**Fig. S1.** Local recruitment of the great tit in relation to synchrony with the caterpillar food peak (synchrony = day of 10 days old young – caterpillar peak day, i.e. chicks are 10 days old at the peak day) in high densities (black lines; 940 individual / study area) and in low densities (red lines; 488 individuals / study area). Other variables were set to average values. Dashed lines indicate 95% confidence intervals. See Table S7 for model parameter coefficients.
