# Supplementary material for: Different Ultimate Factors Define Timing of Breeding in Two Related Species
Source: PLoS One. 2016 Sep 9;11(9):e0162643. doi: 10.1371/journal.pone.0162643 (PMC5017718; doi:10.1371/journal.pone.0162643)
Supplement: S1 Table — (DOCX) [file pone.0162643.s003.docx]

**S1 Table. Summaries of explanatory variables.**

Different ultimate factors define timing of breeding in two related species

Veli-Matti Pakanen, Markku Orell, Emma Vatka, Seppo Rytkönen & Juli Broggi

**Table S1.** Summary of explanatory variables used in the study.

| Variable | Willow tit | | | Great tit | | |
| --- | --- | --- | --- | --- | --- | --- |
|  | mean | min | max | mean | min | max |
| ‘HD’ (days from population average hatching date) | 0.36 | -10.31 | 18.70 | 0.00 | -20.02 | 44.06 |
| Synchrony (days from caterpillar peak) | -7.61 | -25 | 15 | -0.37 | -20 | 46 |
| Peak height (highest caterpillar biomass; g m^-2^) | 0.343 | 0.090 | 0.798 | 0.343 | 0.090 | 0.798 |
| Mass (fledgling mass; g) | 10.97 | 5.5 | 14.5 | 15.67 | 6.1 | 22.3 |
| Density (N individuals in autumn/ study area) | 822 | 524 | 1257 | 730 | 375 | 1055 |
| Distance to center (m) | 2034 | 59 | 4126 | 2092 | 20 | 4592 |
