# Supplementary material for: Different Ultimate Factors Define Timing of Breeding in Two Related Species
Source: PLoS One. 2016 Sep 9;11(9):e0162643. doi: 10.1371/journal.pone.0162643 (PMC5017718; doi:10.1371/journal.pone.0162643)
Supplement: S2 Table — Modelling results for local recruitment of the great tit (Parus major) survival examining the effects of centred hatching date (HD) and synchrony (SYN). Models also include PK = peak height in caterpillar food abundance, DC = distance to the center of the study area, MASS = mass, DEN = density, + additive effects, *interaction and variable name2 = quadratic effect of the variable, k = number of parameters. QAIC is scaled with ĉ = 1.137. Model parameters for survival include the intercept and age, and for recapture rates the intercept, but model names include only the covariates to increase readability. (DOCX) [file pone.0162643.s004.docx]

**S2 Table. Modelling results for local recruitment of great tits.**

Different ultimate factors define timing of breeding in two related species

Veli-Matti Pakanen, Markku Orell, Emma Vatka, Seppo Rytkönen & Juli Broggi

**Table S2.** Modelling results for local recruitment of the great tit (*Parus major*) survival examining the effects of centred hatching date (HD) and synchrony (SYN). Models also include PK = peak height in caterpillar food abundance, DC = distance to the center of the study area, MASS = mass, DEN = density, + additive effects, *interaction and variable name2 = quadratic effect of the variable, k = number of parameters. QAIC is scaled with ĉ = 1.137. Model parameters for survival include the intercept and age, and for recapture rates the intercept, but model names include only the covariates to increase readability.

| # | Model | QAICc | ∆QAICc | QAICc Weights | k |
| --- | --- | --- | --- | --- | --- |
| A1 | DC+MASS+MASS2+DEN+SYN+SYN2+PK+DEN*SYN+DEN*S2 | 2765.58 | 0.00 | 0.241 | 12 |
| A2 | DC+MASS+DEN+SYN+SYN2+DEN*SYN+DEN*SYN2 | 2767.43 | 1.85 | 0.096 | 10 |
| A3 | DC+MASS+MASS2+DEN+SYN+DEN*SYN | 2767.67 | 2.09 | 0.085 | 9 |
| A4 | DC+MASS+MASS2+DEN+SYN+SYN2+PK+DEN*SYN | 2768.19 | 2.60 | 0.066 | 11 |
| A5 | DC+MASS+MASS2+DEN+SYN+DEN*SYN+D*SYN2 | 2768.52 | 2.94 | 0.055 | 10 |
| A6 | DC+MASS+DEN+SYN+SYN2+DEN*SYN | 2769.98 | 4.39 | 0.027 | 9 |
| A7 | DC+MASS+MASS2+DEN+HD+HD2+DEN*HD2 | 2770.20 | 4.61 | 0.024 | 10 |
| A8 | DC+MASS+MASS2+DEN+HD+HD+DC*HD2 | 2770.53 | 4.94 | 0.020 | 10 |
| A9 | DC+MASS+MASS2+HD+HD2+DC*HD2 | 2770.63 | 5.04 | 0.019 | 9 |
| A10 | DC+MASS+MASS2+SYN+SYN2+PK | 2770.91 | 5.32 | 0.017 | 9 |
| A11 | DC+MASS+MASS2+SYN+SYN2 | 2770.93 | 5.34 | 0.017 | 8 |
| A12 | DC+MASS+MASS2+DEN+SYN+SYN2 | 2771.36 | 5.78 | 0.013 | 9 |
| A13 | DC+MASS+MASS2+DEN+HD+HD2+DEN*HD+DEN*HD2 | 2771.58 | 6.00 | 0.012 | 11 |
| A14 | DC+MASS+MASS2+SYN+SYN2+MASS*SYN2 | 2771.68 | 6.09 | 0.011 | 9 |
| A15 | DC+MASS+MASS2+DEN+SYN+DEN*SYN2 | 2771.81 | 6.22 | 0.011 | 9 |
| A16 | DC+MASS+MASS2+SYN+SYN2+PK+MASS*SYN2 | 2771.81 | 6.23 | 0.011 | 10 |
| A17 | DC+MASS+MASS2+SYN+SYN2+PK+DC*SYN2 | 2771.83 | 6.25 | 0.011 | 10 |
| A18 | DC+MASS+MASS2+SYN+SYN2+DC*SYN2 | 2771.84 | 6.25 | 0.011 | 9 |
| A19 | DC+MASS+MASS2+SYN+SYN2+PK+SYN*PK | 2771.89 | 6.31 | 0.010 | 10 |
| A20 | DC+MASS+MASS2+SYN+PK+SYN*PK | 2771.95 | 6.37 | 0.010 | 9 |
| A21 | DC+MASS+MASS2+DEN+SYN+SYN2+PK | 2772.24 | 6.66 | 0.009 | 10 |
| A22 | DC+MASS+MASS2+DEN+HD+HD2 | 2772.34 | 6.75 | 0.008 | 9 |
| A23 | DC+MASS+MASS2+DEN+HD+HD+DC*HD+DC*HD2 | 2772.35 | 6.77 | 0.008 | 11 |
| A24 | DC+MASS+MASS2+HD+HD2+DC*HD+DC*HD2 | 2772.43 | 6.84 | 0.008 | 10 |
| A25 | DC+MASS+MASS2+SYN+SYN2+DC*SYN | 2772.47 | 6.88 | 0.008 | 9 |
| A26 | DC+MASS+DEN+HD+HD2+DEN*HD2 | 2772.48 | 6.90 | 0.008 | 9 |
| A27 | DC+MASS+MASS2+HD+HD2 | 2772.48 | 6.90 | 0.008 | 8 |
| A28 | DC+MASS+MASS2+SYN+SYN2+PK+DC*SYN | 2772.50 | 6.92 | 0.008 | 10 |
| A29 | DC+MASS+MASS2+SYN+SYN2+PK+MASS*SYN | 2772.75 | 7.17 | 0.007 | 10 |
| A30 | DC+MASS+MASS2+SYN+SYN2+MASS*SYN | 2772.76 | 7.18 | 0.007 | 9 |
| A31 | DC+MASS+MASS2+SYN+SYN2+PK+SYN2*PK | 2772.82 | 7.23 | 0.006 | 10 |
| A32 | DC+MASS+MASS2+DEN+SYN+SYN2+PK+SYN*PK | 2772.95 | 7.36 | 0.006 | 11 |
| A33 | DC+MASS+DEN+HD+HD2+DC*HD2 | 2772.95 | 7.37 | 0.006 | 9 |
| A34 | DC+MASS+MASS2+SYN | 2773.07 | 7.48 | 0.006 | 7 |
| A35 | DC+MASS+MASS2+DEN+SYN+SYN2+PK+DC*SYN2 | 2773.21 | 7.63 | 0.005 | 11 |
| A36 | DC+MASS+MASS2+SYN+PK | 2773.26 | 7.67 | 0.005 | 8 |
| A37 | DC+MASS+MASS2+SYN+SYN+MASS*SYN+MASS*SYN2 | 2773.31 | 7.72 | 0.005 | 10 |
| A38 | DC+MASS+SYN+SYN2 | 2773.32 | 7.73 | 0.005 | 7 |
| A39 | DC+MASS+MASS2+DEN+SYN | 2773.33 | 7.75 | 0.005 | 8 |
| A40 | DC+MASS+MASS2+DEN+SYN+SYN2+PK+DEN*SYN2 | 2773.38 | 7.79 | 0.005 | 11 |
| A41 | DC+MASS+MASS2+SYN+SYN2+DC*SYN+DC*SYN2 | 2773.38 | 7.80 | 0.005 | 10 |
| Table continues | | | | | |

Table S2 continues

| # | Model | QAICc | ∆QAICc | QAICc Weights | k |
| --- | --- | --- | --- | --- | --- |
| A42 | DC+MASS+MASS2+SYN+SYN2+PK+MASS*SYN+MASS*SYN2 | 2773.45 | 7.86 | 0.005 | 11 |
| A43 | DC+MASS+MASS2+SYN+SYN2+PK+DC*SYN+DC*SYN2 | 2773.46 | 7.87 | 0.005 | 11 |
| A44 | DC+MASS+DEN+SYN+SYN2 | 2773.78 | 8.20 | 0.004 | 8 |
| A45 | DC+MASS+DEN+HD+HD2+DEN*HD+DEN*HD2 | 2773.82 | 8.23 | 0.004 | 10 |
| A46 | DC+MASS+MASS2+DEN+SYN+SYN2+PK+DC*SYN | 2773.83 | 8.24 | 0.004 | 11 |
| A47 | DC+MASS+SYN+SYN2+PK | 2773.86 | 8.27 | 0.004 | 8 |
| A48 | DC+MASS+MASS2+SYN+SYN2+PK+SYN*PK+SYN2*PK | 2773.90 | 8.32 | 0.004 | 11 |
| A49 | DC+MASS+SYN+SYN2+MASS*SYN2 | 2773.97 | 8.38 | 0.004 | 8 |
| A50 | DC+MASS+MASS2+DEN+HD+HD2+MASS*HD2 | 2773.99 | 8.41 | 0.004 | 10 |
| A51 | DC+MASS+MASS2+DEN+HD+HD2+MASS*HD | 2774.03 | 8.45 | 0.004 | 10 |
| A52 | DC+MASS+MASS2+DEN+SYN+SYN2+PK+SYN2*PK | 2774.14 | 8.55 | 0.003 | 11 |
| A53 | DC+MASS+MASS2+HD+HD2+MASS*HD2 | 2774.21 | 8.62 | 0.003 | 9 |
| A54 | DC+MASS+MASS2+HD+HD2+MASS*HD | 2774.21 | 8.62 | 0.003 | 9 |
| A55 | DC+MASS+MASS2+DEN+HD+HD2+DEN*HD | 2774.33 | 8.75 | 0.003 | 10 |
| A56 | DC+MASS+MASS2+DEN+HD+HD+DC*HD | 2774.34 | 8.76 | 0.003 | 10 |
| A57 | DC+MASS+MASS2+HD+HD2+DC*HD | 2774.49 | 8.90 | 0.003 | 9 |
| A58 | DC+MASS+DEN+HD+HD2 | 2774.69 | 9.10 | 0.003 | 8 |
| A59 | DC+MASS+DEN+HD+HD2+DC*HD+DC*HD2 | 2774.82 | 9.24 | 0.002 | 10 |
| A60 | DC+HD+HD2+MASS | 2774.83 | 9.25 | 0.002 | 7 |
| A61 | DC+MASS+MASS2+DEN+SYN+SYN2+PKDC*SYN+DC*SYN2 | 2774.84 | 9.26 | 0.002 | 12 |
| A62 | DC+MASS+MASS2+DEN+SYN+SYN2+PK+SYN*PK+SYN2*PK | 2774.95 | 9.36 | 0.002 | 12 |
| A63 | DC+MASS+DEN+SYN+SYN2+DEN*SYN2 | 2774.96 | 9.37 | 0.002 | 9 |
| A64 | DC+MASS+DEN+SYN+SYN2+PK | 2775.07 | 9.49 | 0.002 | 9 |
| A65 | DC+MASS+MASS2+SYN+PK+MASS*SYN | 2775.25 | 9.67 | 0.002 | 9 |
| A66 | DC+MASS+SYN+SYN2+MASS*SYN+MASS*SYN2 | 2775.71 | 10.13 | 0.002 | 9 |
| A67 | DC+MASS+MASS2+HD | 2775.73 | 10.14 | 0.002 | 7 |
| A68 | DC+MASS+SYN+SYN2+PK+P*S2 | 2775.79 | 10.21 | 0.001 | 9 |
| A69 | DC+MASS+MASS2+DEN+HD+HD2+MASS*HD+MASS*HD2 | 2775.84 | 10.25 | 0.001 | 11 |
| A70 | DC+MASS+MASS2+DEN+HD | 2775.92 | 10.33 | 0.001 | 8 |
| A71 | DC+MASS+MASS2+HD+HD2+MASS*HD+MASS*HD2 | 2776.06 | 10.47 | 0.001 | 10 |
| A72 | DC+MASS+SYN | 2776.17 | 10.59 | 0.001 | 6 |
| A73 | DC+HD+HD2+MASS+MASS*HD2 | 2776.33 | 10.75 | 0.001 | 8 |
| A74 | DC+MASS+DEN+HD+HD2+MASS*HD | 2776.43 | 10.85 | 0.001 | 9 |
| A75 | DC+MASS+DEN+SYN | 2776.45 | 10.87 | 0.001 | 7 |
| A76 | DC+MASS+DEN+HD+HD2+DEN*HD | 2776.67 | 11.08 | 0.001 | 9 |
| A77 | DC+MASS+DEN+HD+HD2+DC*HD | 2776.69 | 11.10 | 0.001 | 9 |
| A78 | DC+MASS+SYN+SYN2+PK+PK*SYN+PK*SYN2 | 2776.87 | 11.29 | 0.001 | 10 |
| A79 | DC+MASS+SYN+PK | 2776.92 | 11.33 | 0.001 | 7 |
| A80 | DC+MASS+MASS2 | 2777.05 | 11.47 | 0.001 | 6 |
| A81 | DC+MASS+MASS2+DEN | 2777.19 | 11.60 | 0.001 | 7 |
| A82 | DC+MASS+MASS2+DEN+HD+DEN*HD | 2777.73 | 12.15 | 0.001 | 9 |
| A83 | DC+HD+HD2+MASS+MASS*HD+MASS*HD2 | 2778.26 | 12.67 | 0.000 | 9 |
| A84 | DC+HD+MASS | 2778.86 | 13.28 | 0.000 | 6 |
| A85 | DC+MASS+DEN+HD | 2779.08 | 13.50 | 0.000 | 7 |
| A86 | DC+MASS | 2780.37 | 14.78 | 0.000 | 5 |
| A87 | DC+MASS+DEN | 2780.55 | 14.96 | 0.000 | 6 |
| Table continues | | | | | |

Table S2 continues

| # | Model | QAICc | ∆QAICc | QAICc Weights | k |
| --- | --- | --- | --- | --- | --- |
| A88 | DC+SYN+SYN2+PK | 2797.80 | 32.21 | 0.000 | 7 |
| A89 | DC+SYN+PK | 2798.65 | 33.07 | 0.000 | 6 |
| A90 | DC+DEN+SYN+SYN2+PK | 2799.31 | 33.73 | 0.000 | 8 |
| A91 | DC+DEN+SYN+PK | 2800.00 | 34.42 | 0.000 | 7 |
| A92 | DC+HD+HD2+DEN+DEN*HD2 | 2801.10 | 35.52 | 0.000 | 8 |
| A93 | DC+DEN+SYN+SYN2 | 2801.27 | 35.68 | 0.000 | 7 |
| A94 | DC+DEN+SYN | 2801.41 | 35.83 | 0.000 | 6 |
| A95 | DC+SYN+SYN2 | 2801.43 | 35.85 | 0.000 | 6 |
| A96 | DC+SYN | 2801.70 | 36.12 | 0.000 | 5 |
| A97 | DC+HD+HD2+DEN+DEN*HD+DEN*HD2 | 2802.53 | 36.95 | 0.000 | 9 |
| A98 | DC+HD+HD2+DEN | 2802.87 | 37.28 | 0.000 | 7 |
| A99 | DC+HD+HD2 | 2803.56 | 37.98 | 0.000 | 6 |
| A100 | DC+HD+DEN | 2803.62 | 38.04 | 0.000 | 6 |
| A101 | DC+HD | 2804.09 | 38.50 | 0.000 | 5 |
| A102 | DC+DEN | 2805.34 | 39.75 | 0.000 | 5 |
| A103 | DC | 2805.89 | 40.30 | 0.000 | 4 |
| A104 | CONSTANT | 2806.64 | 41.06 | 0.000 | 3 |
