# Supplementary material for: Different Ultimate Factors Define Timing of Breeding in Two Related Species
Source: PLoS One. 2016 Sep 9;11(9):e0162643. doi: 10.1371/journal.pone.0162643 (PMC5017718; doi:10.1371/journal.pone.0162643)
Supplement: S3 Table — Modelling results for local recruitment of the willow tit (Poecile montanus) examining the effects of centred hatching date (HD) and synchrony (SYN). Models also include PK = peak height in caterpillar food abundance, DC = distance to the center of the study area, MASS = mass, DEN = density, + additive effects, *interaction and variable name2 = quadratic effect of the variable. QAIC is scaled with ĉ = 1.039. Model parameters for survival include also the intercept and age, and for recapture rates the intercept and time, but model names include only the covariates to increase readability. (DOCX) [file pone.0162643.s005.docx]

**S3 Table. Modelling results for local recruitment of willow tits.**

Different ultimate factors define timing of breeding in two related species

Veli-Matti Pakanen, Markku Orell, Emma Vatka, Seppo Rytkönen & Juli Broggi

**Table S3.** Modelling results for local recruitment of the willow tit (*Poecile montanus*) examining the effects of centred hatching date (HD) and synchrony (SYN). Models also include PK = peak height in caterpillar food abundance, DC = distance to the center of the study area, MASS = mass, DEN = density, + additive effects, *interaction and variable name2 = quadratic effect of the variable. QAIC is scaled with ĉ = 1.039. Model parameters for survival include also the intercept and age, and for recapture rates the intercept and time, but model names include only the covariates to increase readability.

| # | Model | QAICc | ∆QAICc | QAICc Weight | k |
| --- | --- | --- | --- | --- | --- |
| B1 | DC+DEN+MASS+HD+HD2+DC*HD | 3634.22 | 0.00 | 0.216 | 21 |
| B2 | DC+DEN+MASS+MASS2+HD+HD2+DC*HD | 3634.51 | 0.30 | 0.186 | 22 |
| B3 | DC+DEN+MASS+HD+HD2+DC*HD+DC*HD2 | 3635.57 | 1.35 | 0.110 | 22 |
| B4 | DC+DEN+MASS+MASS2+HD+HD2+DC*HD+DC*HD2 | 3635.90 | 1.69 | 0.093 | 23 |
| B5 | DC+DEN+MASS+HD+HD2 | 3636.97 | 2.75 | 0.055 | 20 |
| B6 | DC+DEN+MASS+MASS2+HD+HD2 | 3637.24 | 3.03 | 0.048 | 21 |
| B7 | DC+DEN+MASS+HD+HD2+DEN*HD2 | 3638.37 | 4.15 | 0.027 | 21 |
| B8 | DC+DEN+MASS+HD+HD2+DC*HD2 | 3638.42 | 4.20 | 0.026 | 21 |
| B9 | DC+DEN+MASS+MASS2+HD+HD2+DEN*HD2 | 3638.64 | 4.42 | 0.024 | 22 |
| B10 | DC+DEN+MASS+HD+HD2+HD*MASS | 3638.66 | 4.45 | 0.023 | 21 |
| B11 | DC+DEN+MASS+MASS2+HD+HD2+DC*HD2 | 3638.73 | 4.51 | 0.023 | 22 |
| B12 | DC+DEN+MASS+MASS2+HD+HD2+MASS*HD | 3638.74 | 4.52 | 0.023 | 22 |
| B13 | DC+DEN+MASS+HD+HD2+DEN*HD | 3638.79 | 4.57 | 0.022 | 21 |
| B14 | DC+DEN+MASS+HD+HD2+MASS*HD2 | 3638.87 | 4.66 | 0.021 | 21 |
| B15 | DC+DEN+MASS+MASS2+HD+HD2+MASS*HD2 | 3639.04 | 4.82 | 0.019 | 22 |
| B16 | DC+DEN+MASS+MASS2+HD+HD2+DEN*HD | 3639.04 | 4.83 | 0.019 | 22 |
| B17 | DC+DEN+MASS+HD+HD2+DEN*HD+DEN*HD2 | 3640.16 | 5.94 | 0.011 | 22 |
| B18 | DC+DEN+MASS+MASS2+HD+HD2+DEN*HD+DEN*HD2 | 3640.41 | 6.20 | 0.010 | 23 |
| B19 | DC+DEN+MASS+MASS2+HD+HD2+MASS*HD+MASS*HD2 | 3640.56 | 6.34 | 0.009 | 23 |
| B20 | DC+DEN+MASS+HD+HD2+MASS*HD+MASS*HD2 | 3640.59 | 6.37 | 0.009 | 22 |
| B21 | DC+DEN+MASS+HD+DC*HD | 3640.69 | 6.47 | 0.008 | 20 |
| B22 | DC+DEN+MASS+HD | 3641.49 | 7.27 | 0.006 | 19 |
| B23 | DC+DEN+HD+MASS+MASS2 | 3641.84 | 7.63 | 0.005 | 20 |
| B24 | DC+DEN+MASS+HD+MASS*HD | 3643.34 | 9.12 | 0.002 | 20 |
| B25 | DC+HD+HD2+MASS+DC*HD+DC*HD2 | 3644.24 | 10.02 | 0.001 | 21 |
| B26 | DC+HD+HD2+MASS | 3645.74 | 11.53 | 0.001 | 19 |
| B27 | DC+MASS+MASS2+HD+HD2 | 3646.16 | 11.95 | 0.001 | 20 |
| B28 | DC+HD+HD2+MASS+DC*HD2 | 3647.37 | 13.16 | 0.000 | 20 |
| B29 | DC+HD+HD2+MASS+MASS*HD2 | 3647.63 | 13.42 | 0.000 | 20 |
| B30 | DC+DEN+HD+HD2 | 3648.39 | 14.17 | 0.000 | 19 |
| B31 | DC+HD+HD2+MASS+MASS*HD+MASS*HD2 | 3649.48 | 15.26 | 0.000 | 21 |
| B32 | DC+DEN+HD+HD2+DEN*HD2 | 3649.80 | 15.59 | 0.000 | 20 |
| B33 | DC+HD+MASS | 3649.87 | 15.65 | 0.000 | 18 |
| B34 | DC+DEN+HD+HD2+DEN*HD | 3650.07 | 15.86 | 0.000 | 20 |
| B35 | DC+MASS+MASS2+HD | 3650.36 | 16.14 | 0.000 | 19 |
| B36 | DC+DEN+HD+HD2+DEN*HD+DEN*HD2 | 3651.45 | 17.24 | 0.000 | 21 |
| B37 | DC+DEN+MASS+SYN*PK+SYN2 | 3652.83 | 18.61 | 0.000 | 22 |
| B38 | DC+DEN+MASS+SYN+SYN2+MASS*SYN2 | 3653.21 | 19.00 | 0.000 | 21 |
| B39 | DC+MASS+MASS2+DEN+SYN+SYN2+PK+SYN*PK | 3653.28 | 19.06 | 0.000 | 23 |
| B40 | DC+DEN+MASS+MASS2+SYN+SYN2+MASS*SYN2 | 3653.36 | 19.14 | 0.000 | 22 |
| B41 | DC+DEN+MASS+SYN+SYN2+PK+SYN*PK+SYN2*PK | 3653.56 | 19.35 | 0.000 | 23 |
| Table continues | | | | | |

Table S3 continues

| # | Model | QAICc | ∆QAICc | QAICc Weight | k |
| --- | --- | --- | --- | --- | --- |
| B42 | DC+DEN+MASS+SYN+SYN2 | 3653.75 | 19.54 | 0.000 | 20 |
| B43 | DC+MASS+MASS2+DEN+SYN+SYN2+PK+SYN*PK+SYN2*PK | 3653.99 | 19.77 | 0.000 | 24 |
| B44 | DC+DEN+MASS+SYN+SYN2+MASS*SYN | 3654.35 | 20.14 | 0.000 | 21 |
| B45 | DC+DEN+MASS+MASS2+SYN+SYN2 | 3654.40 | 20.19 | 0.000 | 21 |
| B46 | DC+DEN+HD | 3654.44 | 20.23 | 0.000 | 18 |
| B47 | DC+DEN+MASS+SYN+SYN2+DEN*SYN2 | 3654.45 | 20.23 | 0.000 | 21 |
| B48 | DC+DEN+MASS+MASS2+SYN+SYN2+MASS*SYN | 3654.51 | 20.30 | 0.000 | 22 |
| B49 | DC+DEN+MASS+SYN+SYN2+MASS*SYN+MASS*SYN2 | 3654.84 | 20.63 | 0.000 | 22 |
| B50 | DC+DEN+MASS+SYN+SYN2*PK | 3654.86 | 20.64 | 0.000 | 22 |
| B51 | DC+DEN+MASS+SYN+SYN2+DC*SYN2 | 3654.90 | 20.68 | 0.000 | 21 |
| B52 | DC+MASS+SYN+SYN2+PK+SYN*PK | 3654.90 | 20.69 | 0.000 | 21 |
| B53 | DC+DEN+MASS+SYN+SYN2+DEN*SYN | 3655.08 | 20.86 | 0.000 | 21 |
| B54 | DC+DEN+MASS+MASS2+SYN+SYN2+MASS*SYN+MASS*SYN2 | 3655.15 | 20.93 | 0.000 | 23 |
| B55 | DC+DEN+MASS+MASS2+SYN+SYN2+DEN*SYN2 | 3655.20 | 20.99 | 0.000 | 22 |
| B56 | DC+DEN+MASS+SYN+SYN2+PK+MASS*SYN2 | 3655.21 | 20.99 | 0.000 | 22 |
| B57 | DC+DEN+MASS+SYN+SYN2+DC*SYN | 3655.22 | 21.01 | 0.000 | 21 |
| B58 | DC+MASS+MASS2+DEN+SYN+SYN2+PK+PK*SYN2 | 3655.36 | 21.15 | 0.000 | 23 |
| B59 | DC+DEN+MASS+MASS2+SYN+SYN2+DC*SYN2 | 3655.53 | 21.32 | 0.000 | 22 |
| B60 | DC+DEN+MASS+SYN+SYN2+PK | 3655.75 | 21.54 | 0.000 | 21 |
| B61 | DC+DEN+MASS+MASS2+SYN+SYN2+DEN*SYN | 3655.81 | 21.59 | 0.000 | 22 |
| B62 | DC+DEN+MASS+MASS2+SYN+SYN2+DC*SYN | 3655.85 | 21.63 | 0.000 | 22 |
| B63 | DC+MASS+SYN+SYN2+PK+PK*SYN+PK*SYN2 | 3655.93 | 21.72 | 0.000 | 22 |
| B64 | DC+DEN+MASS+SYN+SYN2+PK+DEN*SYN2 | 3656.29 | 22.08 | 0.000 | 22 |
| B65 | DC+DEN+MASS+SYN+SYN2+DEN*SYN+DEN*SYN2 | 3656.33 | 22.11 | 0.000 | 22 |
| B66 | DC+DEN+MASS+SYN+SYN2+PK+MASS*SYN | 3656.35 | 22.14 | 0.000 | 22 |
| B67 | DC+MASS+MASS2+DEN+SYN+SYN2+PK | 3656.38 | 22.16 | 0.000 | 22 |
| B68 | DC+MASS+SYN+SYN2+PK+PK*SYN2 | 3656.78 | 22.57 | 0.000 | 21 |
| B69 | DC+DEN+MASS+SYN+SYN2+DC*SYN+DC*SYN2 | 3656.84 | 22.62 | 0.000 | 22 |
| B70 | DC+DEN+MASS+SYN+SYN2+PK+MASS*SYN+MASS*SYN2 | 3656.84 | 22.62 | 0.000 | 23 |
| B71 | DC+DEN+MASS+SYN+SYN2+PK+DC*SYN2 | 3656.89 | 22.68 | 0.000 | 22 |
| B72 | DC+DEN+MASS+SYN+SYN2+PK+DEN*SYN | 3657.00 | 22.78 | 0.000 | 22 |
| B73 | DC+DEN+MASS+MASS2+SYN+SYN2+DEN*SYN+DEN*SYN2 | 3657.07 | 22.85 | 0.000 | 23 |
| B74 | DC+HD+HD2 | 3657.15 | 22.93 | 0.000 | 18 |
| B75 | DC+DEN+MASS+MASS2+SYN+SYN2+DC*SYN+DC*SYN2 | 3657.49 | 23.27 | 0.000 | 23 |
| B76 | DC+DEN+MASS | 3657.66 | 23.44 | 0.000 | 18 |
| B77 | DC+MASS+MASS2+DEN+SYN+SYN2+PK+DC*SYN | 3657.81 | 23.59 | 0.000 | 23 |
| B78 | DC+SYN+SYN2+MASS | 3657.83 | 23.61 | 0.000 | 19 |
| B79 | DC+MASS+SYN+SYN2+PK | 3658.02 | 23.80 | 0.000 | 20 |
| B80 | DC+DEN+MASS+SYN+SYN2+PK+DEN*SYN+DEN*SYN2 | 3658.17 | 23.95 | 0.000 | 23 |
| B81 | DC+DEN+MASS+MASS2 | 3658.33 | 24.12 | 0.000 | 19 |
| B82 | DC+MASS+MASS2+SYN+SYN2+PK | 3658.54 | 24.32 | 0.000 | 21 |
| B83 | DC+MASS+MASS2+SYN+SYN2 | 3658.58 | 24.37 | 0.000 | 20 |
| B84 | DC+DEN+MASS+SYN+SYN2+PK+DC*SYN+DC*SYN2 | 3658.84 | 24.62 | 0.000 | 23 |
| B85 | DC+DEN+MASS+SYN | 3659.62 | 25.40 | 0.000 | 19 |
| B86 | DC+DEN+MASS+MASS2+SYN | 3660.31 | 26.09 | 0.000 | 20 |
| Table continues | | | | | |

Table S3 continues

| # | Model | QAICc | ∆QAICc | QAICc Weight | k |
| --- | --- | --- | --- | --- | --- |
| B87 | DC+HD | 3662.70 | 28.48 | 0.000 | 17 |
| B88 | DC+MASS | 3663.56 | 29.34 | 0.000 | 17 |
| B89 | DC+MASS+MASS2 | 3664.31 | 30.10 | 0.000 | 18 |
| B90 | DC+SYN+MASS | 3665.08 | 30.87 | 0.000 | 18 |
| B91 | DC+MASS+MASS2+SYN | 3665.88 | 31.66 | 0.000 | 19 |
| B92 | DC+MASS+SYN+PK | 3666.26 | 32.04 | 0.000 | 19 |
| B93 | DC+DEN+SYN+SYN2 | 3666.84 | 32.63 | 0.000 | 19 |
| B94 | DC+MASS+MASS2+SYN+PK | 3666.90 | 32.68 | 0.000 | 20 |
| B95 | DC+DEN+SYN+SYN2+PK | 3668.84 | 34.62 | 0.000 | 20 |
| B96 | DC+DEN | 3670.05 | 35.84 | 0.000 | 17 |
| B97 | DC+SYN+SYN2 | 3670.74 | 36.53 | 0.000 | 18 |
| B98 | DC+SYN+SYN2+PK | 3670.96 | 36.74 | 0.000 | 19 |
| B99 | DC+DEN+SYN | 3672.03 | 37.82 | 0.000 | 18 |
| B100 | DC+DEN+SYN+PK | 3673.88 | 39.67 | 0.000 | 19 |
| B101 | DC | 3675.62 | 41.41 | 0.000 | 16 |
| B102 | DC+SYN | 3677.26 | 43.04 | 0.000 | 17 |
| B103 | DC+SYN+PK | 3678.40 | 44.18 | 0.000 | 18 |
| B104 | CONSTANT | 3683.19 | 48.97 | 0.000 | 15 |
