# Supplementary material for: Different Ultimate Factors Define Timing of Breeding in Two Related Species
Source: PLoS One. 2016 Sep 9;11(9):e0162643. doi: 10.1371/journal.pone.0162643 (PMC5017718; doi:10.1371/journal.pone.0162643)
Supplement: S4 Table — Relative importance of variables from models examining the effects of synchrony and hatching date on local recruitment (S2 and S3 Tables) shown by summed model weights (w) and average model weights (n = number of models) for both the great tit and the willow tit. (DOCX) [file pone.0162643.s006.docx]

**S4 Table. Relative importance of variables.**

Different ultimate factors define timing of breeding in two related species

Veli-Matti Pakanen, Markku Orell, Emma Vatka, Seppo Rytkönen & Juli Broggi

**Table S4.** Relative importance of variables from models examining the effects of synchrony and hatching date on local recruitment (Tables S2-S3) shown by summed model weights (w) and average model weights (n = number of models) for both the great tit and the willow tit.

|  | The great tit | | | The willow tit | | |
| --- | --- | --- | --- | --- | --- | --- |
| Model variable | Σ(w) | n | average(w) | Σ(w) | n | average(w) |
| SYN | 0.8318 | 58 | 0.0143 | 0.0003 | 58 | 0.0000 |
| SYN^2^ | 0.7159 | 45 | 0.0159 | 0.0003 | 48 | 0.0000 |
| HD | 0.1664 | 39 | 0.0043 | 0.9997 | 39 | 0.0256 |
| HD^2^ | 0.1664 | 31 | 0.0054 | 0.9783 | 31 | 0.0316 |
| PK | 0.4523 | 32 | 0.0141 | 0.0001 | 28 | 0.0000 |
| MASS | 1.0000 | 87 | 0.0115 | 0.9996 | 86 | 0.0116 |
| MASS2 | 0.8194 | 58 | 0.0141 | 0.4590 | 34 | 0.0135 |
| DEN | 0.7610 | 53 | 0.0144 | 0.9965 | 75 | 0.0133 |
| DC | 1.0000 | 103 | 0.0097 | 1.0000 | 103 | 0.0097 |
| CONSTANT | 0.0000 | 1 | 0.0000 | 0.0000 | 1 | 0.0000 |
| DEN x SYN | 0.5697 | 6 | 0.0949 | 0.0000 | 6 | 0.0000 |
| DEN x SYN2 | 0.4101 | 6 | 0.0684 | 0.0000 | 6 | 0.0000 |
| DEN x HD | 0.0205 | 6 | 0.0034 | 0.0894 | 6 | 0.0149 |
| DEN x HD2 | 0.0477 | 6 | 0.0079 | 0.0640 | 6 | 0.0107 |
| MASS x SYN | 0.0266 | 6 | 0.0044 | 0.0000 | 6 | 0.0000 |
| MASS x SYN2 | 0.0372 | 6 | 0.0062 | 0.0001 | 6 | 0.0000 |
| MASS x HD | 0.0110 | 6 | 0.0018 | 0.0663 | 6 | 0.0111 |
| MASS x HD2 | 0.0111 | 6 | 0.0019 | 0.0589 | 6 | 0.0098 |
| DC x SYN | 0.0312 | 6 | 0.0052 | 0.0000 | 6 | 0.0000 |
| DC x SYN2 | 0.0385 | 6 | 0.0064 | 0.0000 | 6 | 0.0000 |
| DC x HD | 0.0252 | 6 | 0.0042 | 0.6071 | 6 | 0.1012 |
| DC x HD2 | 0.0642 | 6 | 0.0107 | 0.2624 | 6 | 0.0437 |
| PK x SYN | 0.0332 | 6 | 0.0055 | 0.0001 | 6 | 0.0000 |
| PK x SYN2 | 0.0182 | 6 | 0.0030 | 0.0000 | 6 | 0.0000 |
