# Supplementary material for: Different Ultimate Factors Define Timing of Breeding in Two Related Species
Source: PLoS One. 2016 Sep 9;11(9):e0162643. doi: 10.1371/journal.pone.0162643 (PMC5017718; doi:10.1371/journal.pone.0162643)
Supplement: S5 Table — Modelling results for local recruitment of the great tit (Parus major) examining the effects of and synchrony (SYN) when centred hatching date (HD) and its quadratic term (HD2) are included in the best model from S2 Table. Models also include PK = peak height in caterpillar food abundance, DC = distance to the center of the study area, MASS = mass, DEN = density, + additive effects, *interaction and variable name2 = quadratic effect of the variable, k = number of parameters. QAIC is scaled with ĉ = 1.137. Model parameters for survival include the intercept and age, and for recapture rates the intercept, but model names include only the covariates to increase readability. (DOCX) [file pone.0162643.s007.docx]

**S5 Table. Modelling results for local recruitment of the great tit with synchrony and hatching date in the same model.**

Different ultimate factors define timing of breeding in two related species

Veli-Matti Pakanen, Markku Orell, Emma Vatka, Seppo Rytkönen & Juli Broggi

**Table S5.** Modelling results for local recruitment of the great tit (*Parus major*) examining the effects of and synchrony (SYN) when centred hatching date (HD) and its quadratic term (HD2) are included in the best model from Table S2. Models also include PK = peak height in caterpillar food abundance, DC = distance to the center of the study area, MASS = mass, DEN = density, + additive effects, *interaction and variable name2 = quadratic effect of the variable, k = number of parameters. QAIC is scaled with ĉ = 1.137. Model parameters for survival include the intercept and age, and for recapture rates the intercept, but model names include only the covariates to increase readability.

| # | Model | QAICc | ∆QAICc | QAICc Weights | k |
| --- | --- | --- | --- | --- | --- |
| C1 | DC+MASS+MASS2+DEN+SYN+SYN2+PK+DEN*SYN+DEN*SYN2+HD+HD2 | 2764.34 | 0.00 | 0.650 | 14 |
| A1 | DC+MASS+MASS2+DEN+SYN+SYN2+PK+DEN*SYN+DEN*SYN2 | 2765.58 | 1.24 | 0.350 | 12 |
