# Supplementary material for: Different Ultimate Factors Define Timing of Breeding in Two Related Species
Source: PLoS One. 2016 Sep 9;11(9):e0162643. doi: 10.1371/journal.pone.0162643 (PMC5017718; doi:10.1371/journal.pone.0162643)
Supplement: S6 Table — Modelling results for local recruitment of the willow tit (Poecile montanus) examining the effect of synchrony (SYN) when synchrony (SYN) and its quadratic term (SYN) are included in the best model from S3 Table (i.e. a model containing centred hatching date). Models also include DC = distance to the center of the study area, MASS = mass, DEN = density, + additive effects, *interaction and variable name2 = quadratic effect of the variable. QAIC is scaled with ĉ = 1.039. Model parameters for survival include also the intercept and age, and for recapture rates the intercept and time, but model names include only the covariates to increase readability. (DOCX) [file pone.0162643.s008.docx]

**S6 Table. Modelling results for local recruitment of the willow tit with synchrony and hatching date in the same model.**

Different ultimate factors define timing of breeding in two related species

Veli-Matti Pakanen, Markku Orell, Emma Vatka, Seppo Rytkönen & Juli Broggi

**Table S6.** Modelling results for local recruitment of the willow tit (*Poecile montanus*) examining the effect of synchrony (SYN) when synchrony (SYN) and its quadratic term (SYN) are included in the best model from Table S3 (i.e. a model containing centred hatching date). Models also include DC = distance to the center of the study area, MASS = mass, DEN = density, + additive effects, *interaction and variable name2 = quadratic effect of the variable. QAIC is scaled with ĉ = 1.039. Model parameters for survival include also the intercept and age, and for recapture rates the intercept and time, but model names include only the covariates to increase readability.

| # | Model | QAICc | ∆QAICc | QAICc Weights | k |
| --- | --- | --- | --- | --- | --- |
| D1 | DC+DEN+MASS+HD+HD2+DC*HD+SYN+SYN2 | 3631.59 | 0.00 | 0.788 | 23 |
| B1 | DC+DEN+MASS+HD+HD2+DC*HD | 3634.22 | 2.63 | 0.212 | 21 |
