# Supplementary material for: Different Ultimate Factors Define Timing of Breeding in Two Related Species
Source: PLoS One. 2016 Sep 9;11(9):e0162643. doi: 10.1371/journal.pone.0162643 (PMC5017718; doi:10.1371/journal.pone.0162643)
Supplement: S7 Table — The regression coefficients (SE) for the top models (Delta QAICc <2) describing local recruitment of the great tit in relation to synchrony from S5 Table. Coefficients are presented in the logit scale. Variables that had confidence intervals that do not include zero are in bold. (DOCX) [file pone.0162643.s009.docx]

**S7 Table. Regression coefficients on the top models describing great tit local recruitment in relation to synchrony.**

Different ultimate factors define timing of breeding in two related species

Veli-Matti Pakanen, Markku Orell, Emma Vatka, Seppo Rytkönen & Juli Broggi

**Table S7.** The regression coefficients (SE) for the top models (Delta QAICc <2) describing local recruitment of the great tit in relation to synchrony from Table S5. Coefficients are presented in the logit scale. Variables that had confidence intervals that do not include zero are in bold.

| Variable | Model C1 | Model A1 |
| --- | --- | --- |
| INT | -0.0874(0.1036) | -0.0853(0.1037) |
| AGE | **-11.1073(3.3194)** | **-11.2283(3.3303)** |
| DC | -0.0001(0.0001) | **-0.0001(0.0001)** |
| MASS | **0.9831(0.4158)** | **0.9836(0.4172)** |
| MASS2 | **-0.0259(0.013)** | **-0.026(0.0131)** |
| DEN | -0.0007(0.0006) | -0.0004(0.0005) |
| SYN | **0.2111(0.0682)** | **0.1945(0.0671)** |
| SYN2 | **-0.0179(0.0062)** | **-0.0136(0.0053)** |
| PK | **0.0074(0.0036)** | 0.0046(0.0033) |
| DENxSYN | **0.00002(0.00001)** | **0.00002(0.00001)** |
| DENxSYN2 | **-0.0003(0.0001)** | **-0.0003(0.0001)** |
| HD | 0.0342(0.0218) |  |
| HD2 | -0.0022(0.0019) |  |
