# Supplementary material for: Different Ultimate Factors Define Timing of Breeding in Two Related Species
Source: PLoS One. 2016 Sep 9;11(9):e0162643. doi: 10.1371/journal.pone.0162643 (PMC5017718; doi:10.1371/journal.pone.0162643)
Supplement: S8 Table — The regression coefficients (SE) for the top models (Delta QAICc <2) describing local recruitment of the great tit in relation to centred hatching date from S2 Table. Coefficients are presented in the logit scale. Variables that had confidence intervals that do not include zero are in bold. (DOCX) [file pone.0162643.s010.docx]

**S8 Table. Regression coefficients on the top models describing great tit local recruitment in relation to hatching date.**

Different ultimate factors define timing of breeding in two related species

Veli-Matti Pakanen, Markku Orell, Emma Vatka, Seppo Rytkönen & Juli Broggi

**Table S8.** The regression coefficients (SE) for the top models (Delta QAICc <2) describing local recruitment of the great tit in relation to centred hatching date from Table S2. Coefficients are presented in the logit scale. Variables that had confidence intervals that do not include zero are in bold.

| Variable | Model A7 | Model A8 | Model A9 | Model A13 |  |
| --- | --- | --- | --- | --- | --- |
| INT | -0.0865(0.1037) | -0.0865(0.1037) | -0.0872(0.1037) | -0.0865(0.1037) | |
| AGE | **-11.5516(3.3076)** | **-11.9378(3.3068)** | **-11.4422(3.2848)** | **-11.489(3.3083)** | |
| DC | -0.0001(0.0001) | -0.0001(0.0001) | -0.0001(0.0001) | -0.0001(0.0001) | |
| MASS | **0.9663(0.4135)** | **0.9774(0.4138)** | **0.9786(0.4133)** | **0.9627(0.4136)** | |
| MASS2 | -0.0251(0.0129) | **-0.0255(0.0129)** | **-0.0255(0.0129)** | -0.025(0.0129) | |
| DEN | 0.0004(0.0005) | 0.0006(0.0004) |  | 0.0003(0.0005) | |
| HD | -0.0087(0.0147) | -0.0122(0.0148) | -0.0133(0.0148) | 0.0673(0.0979) | |
| HD2 | **-0.017(0.0069)** | 0.0014(0.0018) | 0.0015(0.0018) | **-0.0191(0.0075)** | |
| DENxHD |  |  |  | -0.0001(0.0001) | |
| DENxHD2 | **0.00002(0.00001)** |  |  | **0.000019(0.000008)** | |
| DCxHD2 |  | -0.000002(0.000001) | -0.000002(0.000001) |  |  |
