# Supplementary material for: Different Ultimate Factors Define Timing of Breeding in Two Related Species
Source: PLoS One. 2016 Sep 9;11(9):e0162643. doi: 10.1371/journal.pone.0162643 (PMC5017718; doi:10.1371/journal.pone.0162643)
Supplement: S10 Table — The regression coefficients (SE) for the top models (Delta QAICc <2) describing local recruitment of the willow tit in relation to centred hatching date from S3 Table. Coefficients are presented in the logit scale. Variables that had confidence intervals that do not include zero are in bold. (DOCX) [file pone.0162643.s012.docx]

**S10 Table. Regression coefficients of the top models describing great tit local recruitment in relation to hatching date.**

Different ultimate factors define timing of breeding in two related species

Veli-Matti Pakanen, Markku Orell, Emma Vatka, Seppo Rytkönen & Juli Broggi

**Table S10.** The regression coefficients (SE) for the top models (Delta QAICc <2) describing local recruitment of the willow tit in relation to centred hatching date from Table S3. Coefficients are presented in the logit scale. Variables that had confidence intervals that do not include zero are in bold.

| Variable | Model B1 | Model B2 | Model B3 | Model B4 |  |
| --- | --- | --- | --- | --- | --- |
| INT | **0.5193(0.0879)** | **0.519(0.0879)** | **0.5193(0.0879)** | **0.519(0.0879)** | |
| AGE | **-4.8255(0.846)** | -12.5712(6.5304) | **-4.8791(0.8487)** | -12.5241(6.4994) | |
| DC | **-0.0003(0.0001)** | **-0.0003(0.0001)** | **-0.0002(0.0001)** | **-0.0002(0.0001)** | |
| DEN | **-0.001(0.0003)** | **-0.001(0.0003)** | **-0.001(0.0003)** | **-0.001(0.0003)** | |
| HD | 0.0277(0.0469) | 0.0268(0.0469) | 0.0252(0.0463) | 0.0245(0.0463) | |
| HD2 | **-0.0091(0.0035)** | **-0.0092(0.0035)** | -0.0037(0.0071) | -0.0039(0.0071) | |
| MASS | **0.2459(0.0698)** | 1.6771(1.1922) | **0.2463(0.0698)** | 1.6592(1.1868) | |
| MASS2 |  | -0.0656(0.0543) |  | -0.0648(0.0541) | |
| DC x HD | **-0.00005(0.00002)** | **-0.00005(0.00002)** | **-0.00005(0.00002)** | **-0.000051(0.000023)** | |
| DC x HD2 | **0.5193(0.0879)** |  | **0.5193(0.0879)** | -0.000003(0.000004) | |
