# Supplementary material for: Different Ultimate Factors Define Timing of Breeding in Two Related Species
Source: PLoS One. 2016 Sep 9;11(9):e0162643. doi: 10.1371/journal.pone.0162643 (PMC5017718; doi:10.1371/journal.pone.0162643)
Supplement: S11 Table — Modelling results for local recruitment of the great tit (Parus major) examining the effects of available caterpillar biomass (BM1 = during ages 0–18; BM2 = during ages 8–13; BM3 = during ages 18–25). The models also include HD = the centred hatching date, DC = distance to the center of the study area, MASS = mass, DEN = density, + additive effects, *interaction and variable name2 = quadratic effect of the variable. QAIC is scaled with ĉ = 1.27. Model parameters for survival include also the intercept and age, and for recapture rates the intercept and time, but model names include only the covariates to increase readability. (DOCX) [file pone.0162643.s013.docx]

**S11 Table. Modelling results for local recruitment of the great tit in relation to available caterpillar biomass.**

Different ultimate factors define timing of breeding in two related species

Veli-Matti Pakanen, Markku Orell, Emma Vatka, Seppo Rytkönen & Juli Broggi

**Table S11.** Modelling results for local recruitment of the great tit (*Parus major*) examining the effects of available caterpillar biomass (BM1 = during ages 0-18; BM2 = during ages 8-13; BM3 = during ages 18-25). The models also include HD = the centred hatching date, DC = distance to the center of the study area, MASS = mass, DEN = density, + additive effects, *interaction and variable name2 = quadratic effect of the variable. QAIC is scaled with ĉ = 1.27. Model parameters for survival include also the intercept and age, and for recapture rates the intercept and time, but model names include only the covariates to increase readability.

| # | Model | QAICc | ∆QAICc | QAICc  Weights | k |
| --- | --- | --- | --- | --- | --- |
| E1 | DC+MASS+MASS2+DEN+HD+HD2+BM3 | 2157.56 | 0.00 | 0.638 | 10 |
| E2 | DC+MASS+ MASS2+DEN+BM3 | 2159.56 | 2.00 | 0.235 | 8 |
| E3 | DC+MASS+ MASS2+DEN+BM1 | 2163.08 | 5.52 | 0.040 | 8 |
| E4 | DC+MASS+ MASS2+DEN+HD+ HD2+BM1 | 2164.07 | 6.51 | 0.025 | 10 |
| E5 | DC+MASS+ MASS2+DEN+BM2 | 2164.44 | 6.88 | 0.020 | 8 |
| E6 | DC+MASS+ MASS2+DEN | 2164.78 | 7.22 | 0.017 | 7 |
| E7 | DC+MASS+ MASS2+DEN+HD+ HD2 | 2165.47 | 7.91 | 0.012 | 9 |
| E8 | DC+MASS+ MASS2+DEN+HD+ HD2+BM2 | 2165.55 | 7.99 | 0.012 | 10 |
