# Supplementary material for: Different Ultimate Factors Define Timing of Breeding in Two Related Species
Source: PLoS One. 2016 Sep 9;11(9):e0162643. doi: 10.1371/journal.pone.0162643 (PMC5017718; doi:10.1371/journal.pone.0162643)
Supplement: S12 Table — Modelling results for local recruitment of the willow tit (Poecile montanus) survival examining the effects of available caterpillar biomass (BM1 = during ages 0–18; BM2 = during ages 8–13; BM3 = during ages 18–25). The models also include HD = the centred hatching date, DC = distance to the center of the study area, MASS = mass, DEN = density, + additive effects, *interaction and variable name2 = quadratic effect of the variable. QAIC is scaled with ĉ = 1.03. Model parameters for survival include also the intercept and age, and for recapture rates the intercept and time, but model names include only the covariates to increase readability. (DOCX) [file pone.0162643.s014.docx]

**S12 Table. Modelling results for local recruitment of the willow tit in relation to available caterpillar biomass.**

Different ultimate factors define timing of breeding in two related species

Veli-Matti Pakanen, Markku Orell, Emma Vatka, Seppo Rytkönen & Juli Broggi

**Table S12.** Modelling results for local recruitment of the willow tit (*Poecile montanus*) survival examining the effects of available caterpillar biomass (BM1 = during ages 0-18; BM2 = during ages 8-13; BM3 = during ages 18-25). The models also include HD = the centred hatching date, DC = distance to the center of the study area, MASS = mass, DEN = density, + additive effects, *interaction and variable name2 = quadratic effect of the variable. QAIC is scaled with ĉ = 1.03. Model parameters for survival include also the intercept and age, and for recapture rates the intercept and time, but model names include only the covariates to increase readability.

| # | Model | QAICc | ∆QAICc | QAICc Weights | k |
| --- | --- | --- | --- | --- | --- |
| F1 | DC+DEN+MASS+HD+HD2+BM1 | 3542.40 | 0.00 | 0.746 | 21 |
| F2 | DC+DEN+MASS+HD+HD2+BM2 | 3544.60 | 2.20 | 0.249 | 21 |
| F3 | DC+DEN+MASS+HD+HD2+BM3 | 3553.35 | 10.95 | 0.003 | 21 |
| F4 | DC+DEN+MASS+HD+HD2 | 3554.60 | 12.20 | 0.002 | 20 |
| F5 | DC+DEN+MASS+BM3 | 3577.79 | 35.39 | 0.000 | 19 |
| F6 | DC+DEN+MASS | 3578.06 | 35.66 | 0.000 | 18 |
| F7 | DC+DEN+MASS+BM1 | 3579.15 | 36.75 | 0.000 | 19 |
| F8 | DC+DEN+MASS+BM2 | 3579.82 | 37.42 | 0.000 | 19 |
