# Supplementary material for: Different Ultimate Factors Define Timing of Breeding in Two Related Species
Source: PLoS One. 2016 Sep 9;11(9):e0162643. doi: 10.1371/journal.pone.0162643 (PMC5017718; doi:10.1371/journal.pone.0162643)
Supplement: S13 Table — The regression coefficients (β) and SE for models describing local recruitment of the great tit derived from models E1, E3 and E5 in S11 Table. Coefficients are presented in the logit scale. Variables that had confidence intervals that do not include zero are in bold. (DOCX) [file pone.0162643.s015.docx]

**S13 Table. Regression coefficients of the top models describing great tit local recruitment in relation to caterpillar biomass.**

Different ultimate factors define timing of breeding in two related species

Veli-Matti Pakanen, Markku Orell, Emma Vatka, Seppo Rytkönen & Juli Broggi

**Table S13.** The regression coefficients (β) and SE for models describing local recruitment of the great tit derived from models E1, E3 and E5 in Table S11. Coefficients are presented in the logit scale. Variables that had confidence intervals that do not include zero are in bold.

|  | E1 | | E3 | | E5 | |
| --- | --- | --- | --- | --- | --- | --- |
| Variable | β | SE | β | SE | β | SE |
| INT | -0.10671 | 0.11795 | -0.10311 | 0.11801 | -0.10252 | 0.11805 |
| AGE | **-10.35078** | **3.49270** | **-10.82651** | **3.49514** | **-10.82077** | **3.49636** |
| DC | -0.00013 | 0.00007 | -0.00012 | 0.00007 | -0.00012 | 0.00007 |
| DEN | 0.00064 | 0.00055 | **0.00109** | **0.00055** | **0.00114** | **0.00056** |
| MASS | 0.79661 | 0.44319 | 0.81341 | 0.44261 | 0.81634 | 0.44310 |
| MASS2 | -0.02114 | 0.01402 | -0.02188 | 0.01399 | -0.02190 | 0.01402 |
| HD | 0.00754 | 0.02475 |  |  |  |  |
| HD2 | -0.00532 | 0.00308 |  |  |  |  |
| BM1 |  |  | 0.01094 | 0.00567 |  |  |
| BM2 |  |  |  |  | 0.00634 | 0.00411 |
| BM3 | **0.01659** | **0.00517** |  |  |  |  |
