# Supplementary material for: Different Ultimate Factors Define Timing of Breeding in Two Related Species
Source: PLoS One. 2016 Sep 9;11(9):e0162643. doi: 10.1371/journal.pone.0162643 (PMC5017718; doi:10.1371/journal.pone.0162643)
Supplement: S14 Table — The regression coefficients (β) and SE for models describing local recruitment of the willow tit derived from models F1, F2 and F3 in S12 Table. Coefficients are presented in the logit scale. Variables that had confidence intervals that do not include zero are in bold. (DOCX) [file pone.0162643.s016.docx]

**S14 Table. Regression coefficients of the top models describing willow tit local recruitment in relation to caterpillar biomass.**

Different ultimate factors define timing of breeding in two related species

Veli-Matti Pakanen, Markku Orell, Emma Vatka, Seppo Rytkönen & Juli Broggi

**Table S14**. The regression coefficients (β) and SE for models describing local recruitment of the willow tit derived from models F1, F2 and F3 in Table S12. Coefficients are presented in the logit scale. Variables that had confidence intervals that do not include zero are in bold.

|  | F1 | | F2 | | F3 | |
| --- | --- | --- | --- | --- | --- | --- |
| Variable | β | SE | β | SE | β | SE |
| INT | **0.49146** | **0.08869** | **0.48812** | **0.08877** | **0.50655** | **0.08878** |
| AGE | **-4.79355** | **0.86456** | **-4.72662** | **0.86064** | **-4.71396** | **0.86267** |
| DC | **-0.00023** | **0.00007** | **-0.00023** | **0.00007** | **-0.00023** | **0.00007** |
| DEN | **-0.00130** | **0.00031** | **-0.00118** | **0.00030** | **-0.00130** | **0.00035** |
| MASS | **0.22172** | **0.07125** | **0.21817** | **0.07117** | **0.24262** | **0.07082** |
| BM1 | **0.00283** | **0.00075** |  |  |  |  |
| BM2 |  |  | **0.00203** | **0.00058** |  |  |
| BM3 |  |  |  |  | 0.00066 | 0.00036 |
| HD | **-0.11448** | **0.02313** | **-0.11239** | **0.02340** | **-0.07715** | **0.02050** |
| HD2 | **-0.00918** | **0.00372** | **-0.01022** | **0.00382** | **-0.00994** | **0.00385** |
